# Fechamento Percutâneo do Canal Arterial em Pacientes Prematuros Abaixo de 2 Kg: Experiência Inicial Brasileira

**DOI:** 10.36660/abc.20210818

**Published:** 2022-08-25

**Authors:** João Luiz Langer Manica, Juliana Rodrigues Neves, Raul Arrieta, Pedro Abujamra, Raul Ivo Rossi, Luiz Carlos Giuliano, Germana Coimbra, Pablo Tomé Teixeirense, João Henrique Aramayo Rossi, Rodrigo Nieckel da Costa, Salvador André Bavaresco Cristóvão, Carlos Pedra

**Affiliations:** 1 Instituto de Cardiologia Fundação Universitária de Cardiologia Porto Alegre RS Brasil Instituto de Cardiologia/Fundação Universitária de Cardiologia (IC/FUC), Porto Alegre, RS – Brasil; 2 Real Hospital Português de Beneficência em Pernambuco Recife PE Brasil Real Hospital Português de Beneficência em Pernambuco, Recife, PE – Brasil; 3 Hospital Sepaco São Paulo SP Brasil Hospital Sepaco, São Paulo, SP – Brasil; 4 Santa Casa de São José dos Campos São José dos Campos SP Brasil Santa Casa de São José dos Campos, São José dos Campos, SP – Brasil; 5 Hospital de Clínicas UNICAMP Campinas SP Brasil Hospital de Clínicas da UNICAMP, Campinas, SP – Brasil; 6 Universidade Federal de Santa Catarina Florianópolis SC Brasil Universidade Federal de Santa Catarina, Florianópolis, SC – Brasil; 7 Hospital SOS Cárdio Florianópolis SC Brasil Hospital SOS Cárdio, Florianópolis, SC – Brasil; 8 Hospital Unimed de Piracicaba Piracicaba SP Brasil Hospital Unimed de Piracicaba, Piracicaba, SP – Brasil; 9 Pontifícia Universidade Católica do Rio Grande do Sul Porto Alegre RS Brasil Pontifícia Universidade Católica do Rio Grande do Sul, Porto Alegre, RS – Brasil; 10 Instituto Dante Pazzanese de Cardiologia São Paulo SP Brasil Instituto Dante Pazzanese de Cardiologia, São Paulo, SP – Brasil; 11 Hospital e Maternidade Santa Joana São Paulo SP Brasil Hospital e Maternidade Santa Joana, São Paulo, SP – Brasil; 12 Beneficência Portuguesa de São Paulo São Paulo SP Brasil Beneficência Portuguesa de São Paulo, São Paulo, SP – Brasil; 13 Hospital do Coração São Paulo SP Brasil Hospital do Coração, São Paulo, SP – Brasil

**Keywords:** Cardiopatias Congênitas, Canal Arterial, Cateterismo, Recém Nascido, Prematuro, Neonatologia

## Abstract

**Fundamento:**

A incidência de *ductus arteriosus* patente (PCA) pode chegar a 50% em pacientes prematuros. Quando hemodinamicamente significativo, pode ser responsável por tempo de ventilação mecânica prolongado, além de importante fator de risco para o aparecimento de enterocolite necrotizante, hemorragia intraventricular e displasia broncopulmonar nessa população.

**Objetivo:**

O objetivo deste estudo é descrever a experiência inicial do fechamento percutâneo de canal arterial em prematuros pesando menos de 2 kg.

**Métodos:**

Trata-se de estudo prospectivo que compreendeu 14 pacientes consecutivos submetidos a fechamento percutâneo de canal arterial de março de 2020 a fevereiro de 2021 em 6 instituições no Brasil.

**Resultados:**

A idade gestacional média ao nascimento foi de 28,45 ±3,14 semanas, a idade média no momento do procedimento foi de 38,85 ±17,35 dias e o peso médio de 1,41±0,41 kg. Dentre os prematuros, 79% necessitavam de ventilação mecânica e 79% tinham feito uso de, em média, 1,5 ciclos de anti-inflamatórios não esteroides. A maioria dos pacientes teve melhora dos parâmetros ventilatórios e o tempo médio de extubação foi de 12,6 ±7,24 dias. A taxa de sucesso foi de 100%. Não houve mortalidade relacionada ao procedimento.

**Conclusão:**

Este estudo concluiu que o fechamento percutâneo do canal arterial em prematuros é uma realidade no Brasil, com resultados satisfatórios e baixa taxa de complicações.

## Introdução

A incidência de *ductus arteriosus* patente (PCA) pode chegar a 50% em pacientes prematuros. Quando hemodinamicamente significativo, pode ser responsável por tempo de ventilação mecânica prolongado, além de importante fator de risco para o aparecimento de enterocolite necrotizante, hemorragia intraventricular e displasia broncopulmonar nessa população,^1 - 5^ Alguns pacientes se beneficiam do fechamento do PCA nesse período da vida com importante progressão no desmame ventilatório e melhora do desfecho global. Historicamente, o tratamento padrão ouro é a terapia medicamentosa com anti-inflamatórios não esteroides, ainda que com taxas de sucesso em torno de 60%, e associada a efeitos adversos significativos.^6^ A ligadura cirúrgica é uma alternativa aos pacientes que não têm condições de dieta enteral ou após falha da terapêutica medicamentosa, entretanto, até 45% dos pacientes desenvolvem instabilidade hemodinâmica logo após o procedimento cirúrgico.^7 - 10^ Até 2010, apenas alguns casos isolados de fechamento percutâneo de canal arterial em prematuros tinham sido reportados na literatura. O advento do dispositivo Amplatzer Duct Occluder II Additional Sizes (ADO II AS) (Abbot Structural Heart, Plymouth, MN) revolucionou o tratamento do PCA em pacientes prematuros com menos de 2 kg e, mais recentemente, o dispositivo Piccolo^tm^(Abbot Structural Heart, Plymouth, MN) foi especificamente desenhado para essa população e aprovado pelo FDA. O objetivo deste estudo é descrever a experiência inicial do fechamento percutâneo de canal arterial em prematuros pesando menos de 2 kg.

## Métodos

Trata-se de estudo prospectivo sobre o tratamento percutâneo do canal arterial em neonatos prematuros com ≤2 kg de peso, realizado com novos dispositivos dedicados a essa população. Os procedimentos foram realizados em 6 centros com operadores distintos no período de março de 2020 a fevereiro de 2021. Todos concordaram em participar deste estudo. Os pacientes foram selecionados a partir de critérios específicos de cada centro envolvido no estudo. Entretanto, todos os pacientes com possibilidade de nutrição enteral haviam recebido tratamento via oral para o fechamento do canal arterial com pelo menos um ciclo de anti-inflamatório não esteroide sem sucesso antes da indicação do procedimento percutâneo. Além disso, a necessidade de ventilação mecânica prolongada refratária associada à presença de canal arterial patente com sinais de sobrecarga volumétrica e aumento atrial esquerdo foi a principal indicação para uso de anti-inflamatórios não esteroides e posteriormente o fechamento percutâneo do canal arterial, em caso de falha do tratamento clínico.

Foram coletados: dados demográficos: idade gestacional, peso de nascimento, gênero, idade (em dias) e peso (em g) no momento do procedimento; dados clínicos: uso de ventilação mecânica e drogas vasoativas, comorbidades associadas, uso de medicações prévias para fechamento do canal arterial (ibuprofeno, paracetamol ou outros) e dados do procedimento: via de acesso vascular, tipo e tamanho do dispositivo, uso de contraste, dificuldades técnicas relatadas e complicações relacionadas, tais como: estenose de artéria pulmonar ou de aorta, entre outras. Dados pós-procedimentos e evolução, como presença de shunt residual, desmame de ventilação mecânica e de drogas vasoativas, e função cardíaca também foram registrados.

As variáveis quantitativas de distribuição normal serão descritas após sua análise, apresentando-se as médias e os desvios-padrão. As variáveis categóricas serão descritas por meio de suas frequências absolutas (n) e relativas (%).

O presente estudo foi aprovado pelo Comitê de Ética em Pesquisa do Instituto de Cardiologia do RS e está em conformidade com a resolução 466/2012. Todos os responsáveis legais dos pacientes assinaram termo de consentimento livre e esclarecido.

### Descrição do dispositivo e técnica

**Figura 1:**
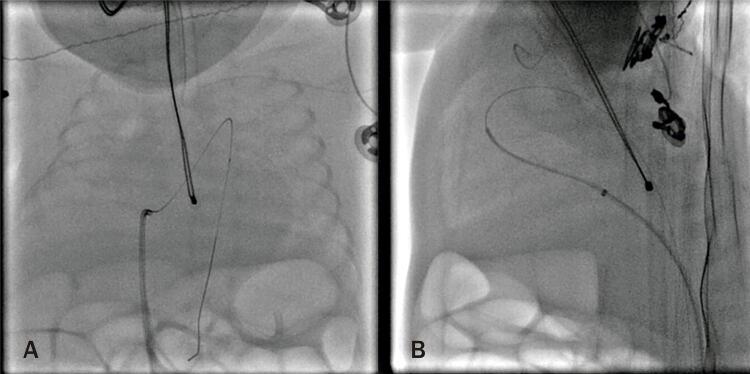
A) Guia 0`014”cruzando canal arterial inserido através de cateter posicionado no ventrículo direito. B) Microcateter sobre guia 0`014”cruzando o canal arterial e servindo de suporte para subida de sistema de liberação do dispositivo.

**Figura 2:**
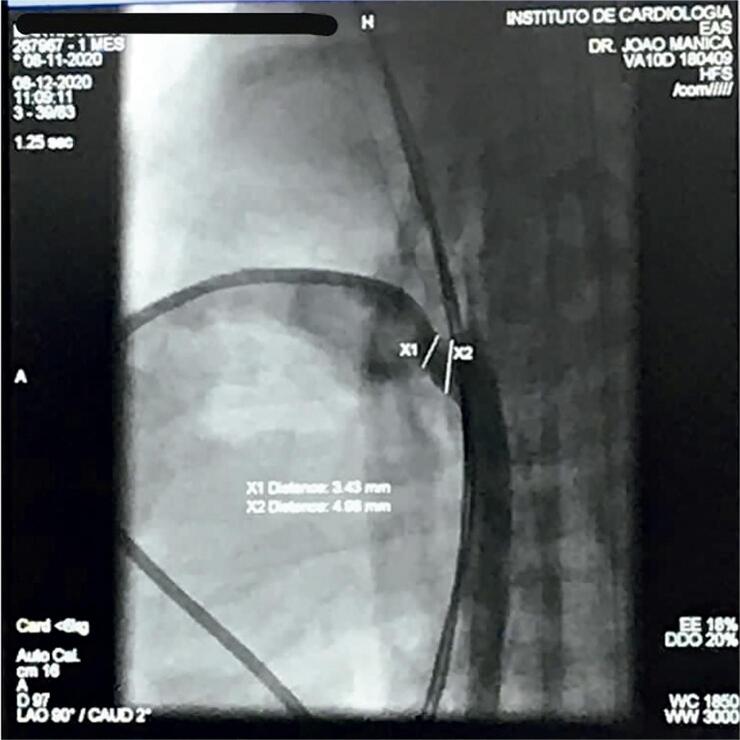
Injeção de contraste em posição 90o através do sistema de liberação.

**Figura 3:**
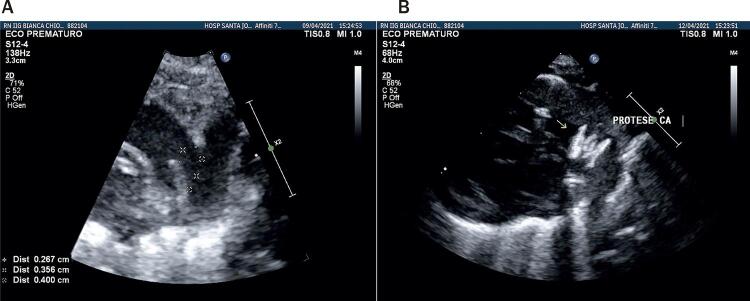
A) Ecocardiograma realizado durante o procedimento com medidas dos diâmetros nas extremidades aórtica e pulmonar e comprimento do canal. B) Ecocardiograma realizado imediatamente após a liberação do dispositivo para descartar lesões residuais como estenoses de artéria pulmonar esquerda ou aorta.

Todos os procedimentos foram realizados no laboratório de cateterismo ou no bloco cirúrgico com arco em C dos respectivos serviços, demandando transporte do neonato para aquele setor. O procedimento é realizado sob anestesia geral. Cuidados com manutenção de temperatura foram tomados com pequenas variações entre centros e, no geral, envolvendo uso de colchão ou mantas aquecidas, monitorização de temperatura com termômetro retal ou esofágico e/ou aquecimento extra com envolvimento de extremidades e polo cefálico. A fim de diminuir o tempo do procedimento, perda sanguínea e administração de fluidos desnecessários, aferições invasivas de pressão não foram rotineiramente realizadas. Foi realizada punção de veia femoral com agulha de 21G ou jelco de 22G e, guiado por ultrassonografia vascular, foi então inserido introdutor radial 4F. Um cateter (4F) de curva JR ou vertebral (Cordis ou Terumo) guiado por guia flexível 0,014” de moderado suporte foi então posicionado através das câmaras direitas e do canal arterial na aorta descendente ( Figura 1A ). Para posicionamento do sistema de liberação da prótese (4Fr TorqVue, Abbot Structural Heart) podem ser utilizadas diferentes técnicas de suporte, a saber: uso de microcateter sobre guia 0,014” anteriormente posicionado em aorta descendente ( Figura 1B ), troca do guia 0,014” por guia 0.035” teflonado e posicionado em aorta descendente, uso do próprio guia 0,014” posicionado em artéria femoral contralateral e pressionado externamente, ou uso de dois guias 0,014” em paralelo, para aumentar o suporte. A fim de preservar a função renal de bebês tão imaturos, o uso de contraste foi limitado a pequenas injeções manuais, apenas se necessário, para elucidação de dúvidas durante o procedimento em alguns casos ( Figura 2 ). Todos os procedimentos foram guiados por ecocardiografia transtorácica tanto para mensuração do canal arterial quanto para posicionamento e liberação do dispositivo ( Figura 3A e B ).

**Figura 4:**
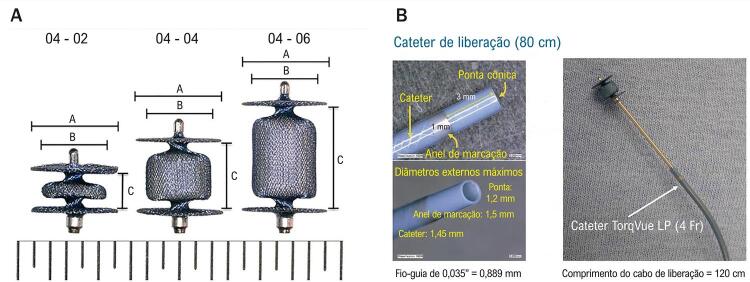
A) Sistema de liberação TorqueVue 4F e dispositivo. B) Dispositivo PiccoloTM de 4 mm de diâmetro nos diferentes comprimentos 2, 4 e 6 mm.

Foram utilizados os dispositivos Amplatzer ADO IIAS e Piccolo (Abbot Structural Heart, Plymouth, MN) em todos os procedimentos, ambos desenvolvidos para oclusão de canal arterial em crianças pequenas, sendo o último específico para neonatos prematuros com peso acima de 700g e disponível comercialmente no Brasil desde meados de 2020. Ambos apresentam características semelhantes em termos de estrutura, sendo compostos de malha de nitinol compacta para minimizar o shunt residual imediatamente após o implante, desenho simétrico composto de dois discos articulados e uma cintura central que corresponde às medidas do dispositivo de baixo perfil com comprimentos de 2, 4 e 6 mm, além de sistema de entrega e cabo também flexíveis para facilitar o posicionamento e a liberação ( Figuras 4A e 4B ). Os dispositivos foram selecionados para ser pelo menos 1 mm maior que o canal arterial em diâmetro e com comprimento menor que o do canal arterial, a fim de evitar estenoses em artéria pulmonar ou aorta. Como já comentado, posicionamento e liberação da prótese foram guiados por ecocardiografia, além da fluoroscopia, observando a presença de shunt residual ou estenoses em ramo esquerdo de artéria pulmonar ou aorta provocadas pelo dispositivo. Se presentes, o dispositivo pode ainda ser reposicionado antes de sua completa liberação. Após o procedimento, os pacientes são transportados em incubadora aquecida de volta à UTI neonatal.

## Resultados

De março de 2020 a fevereiro de 2021, o fechamento percutâneo de canal arterial em prematuros abaixo de 2 kg foi realizado em 14 pacientes por 8 operadores diferentes no Brasil. Dados demográficos estão descritos na Tabela 1 . A idade média dos pacientes no momento do procedimento foi de 38,85±17,35 dias e o peso médio durante o procedimento foi de 1,41±0,41 kg. Quatro pacientes tinham peso < 1 kg no momento do procedimento. A imensa maioria dos pacientes necessitava de ventilação mecânica durante o procedimento (11/14) e pelo menos 6 pacientes tinham diagnóstico de displasia broncopulmonar. Três pacientes não receberam ciclo de ibuprofeno previamente, um devido à insuficiência renal aguda e anúria, um por atresia de duodeno e outro por fístula traqueoesofágica. A indicação do fechamento do canal arterial foi definida pela equipe de neonatologia de cada instituição. Dados sobre o procedimento estão descritos na Tabela 2 . O diâmetro médio do canal arterial mediu em torno de 3,0±0,67 mm e o comprimento médio foi de 6,9±2,12 mm. Nenhum paciente foi submetido a punção arterial. Heparinização após a punção venosa não foi realizada de forma rotineira e depende da escolha do operador. O dispositivo mais utilizado foi o 0402 ADO II AS ou Piccolo (Abbot Structural Heart, Plymouth, MN), em 7 casos, seguido pelo 0502 ADO II AS ou Piccolo (Abbot Structural Heart, Plymouth, MN), em 5 pacientes. Em 1 paciente foi implantado o dispositivo ADO II AS 0504 (Abbot Structural Heart, Plymouth, MN) e, em outro, o dispositivo ADO II AS 0406 (Abbot Structural Heart, Plymouth, MN), ambos mais longos que os demais. Um paciente necessitou de valvoplastia pulmonar durante o procedimento por estenose valvar pulmonar. Dois pacientes apresentaram queda da saturação sistêmica provavelmente relacionada a insuficiência tricúspide durante o procedimento. Nenhum paciente apresentou regurgitação tricúspide significativa após o procedimento. A taxa de sucesso do procedimento foi de 100%. Dois pacientes apresentaram shunt residual imediatamente ao procedimento, porém, em 100% dos casos não havia shunt residual em 7 dias. Os três pacientes dependentes de oxigênio por cateter nasal suspenderam a utilização em uma média de 3 dias após o procedimento. Dentre os pacientes em uso de oxigênio por cateter, um deles teve indicação de fechamento do canal arterial por insuficiência cardíaca franca, com peso menor que o de nascimento após 55 dias de vida, em uso de sonda nasoenteral e quadro clínico de desnutrição importante. Esse paciente teve melhora imediata com retorno à via oral imediatamente após a extubação e alta após 48 horas, em excelente condição clínica. Entre os 11 pacientes em ventilação mecânica, nove pacientes foram extubados em uma média de 13,6±7,4 dias após o procedimento. Não foram relatadas complicações relacionadas ao acesso vascular. Houve 3 óbitos entre os pacientes do estudo, nenhum relacionado ao procedimento. Um paciente melhorou a função renal, teve retorno da diurese 2 dias após o procedimento, melhora dos parâmetros ventilatórios, porém não evoluiu para extubação devido a uma ampla comunicação interventricular e síndrome de Edwards diagnosticada 13 dias após o procedimento. Esse paciente evoluiu a óbito por sepse não relacionada ao procedimento após 22 dias. Outro paciente, com síndrome genética associada, displasia broncopulmonar e hipertensão arterial pulmonar grave, também não atingiu condições clínicas para extubação e evoluiu a óbito 30 dias após o procedimento, por sepse. O outro paciente foi extubado 23 dias após o procedimento, teve ótima evolução e, 57 dias após o procedimento, com aproximadamente 2 kg, teve infecção por SARS-CoV-2 (COVID-19), tendo evoluído novamente para ventilação mecânica e extubado após 10 dias. Após 80 dias do procedimento, pesando em torno de 2300 g, em uso de O_2_ por cateter nasal a 0,5L/min por displasia broncopulmonar, esse paciente apresentou quadro de isquemia mesentérica súbita sendo submetido a cirurgia de urgência e evoluindo para óbito, não sendo possível descartar quadro de trombose pós-COVID-19.

**Tabela 1:** Dados demográficos

IG (semanas)	PN	Idade (dias)	Peso (kg)	VM^*^	DVA^†^	Ciclos de AINES^‡^	Comorbidades
28,6	0,58	62	0,965	Sim	Não	1	BDP^§^, HIC^//^
29,2	1,01	15	0,92	Sim	Sim	Não	IRA^¶^ em anúria, Síndrome genética^1^, ampla CIV^#^
26,6	0,82	41	1,15	Sim	Não	2	BDP, HIC, exposto B24
32	1,55	33	1,85	Sim	Não	2	suspeita de dandy-walker
26	0,85	45	1,55	Sim	Não	3	DBP
27	0,7	28	1,2	Sim	Não	2	
26	0,9	32	1,5	Sim	Não	2	^**^EPV^2^
25	0,8	45	1,2	Sim	Sim	2	DBP
35	2	55	1,95	Não	Não	1	
35	1,2	58	2,0	Não	Não	1	
28	0,8	45	1,45	Sim	Sim	1	BDP, HP^††^ grave, Síndrome genética
27	0,98	7	0,98	Sim	Não	Não	atresia duodeno
26	0,58	63	2,0	Não	Não	2	BDP
27	0,78	15	0,96	Sim	Sim	Não	fístula traqueo esofágica, gastrostomia
28,46	0,97	38,86	1,41	79%	28%	1,55	

IG: Idade gestacional; PN: Peso de nascimento; ^*^VM: ventilação mecânica; ^†^DVA: droga vasoativa; ^‡^AINE: anti-inflamatório não esteroide; ^§^BDP: broncodisplasia pulmonar; ^//^HIC: hemorragia intracraniana; ^¶^IRA: insuficiência renal aguda; ^#^CIV: comunicação interventricular; ^**^EPV: estenose pulmonar valvar; ^††^HP: hipertensão pulmonar.^1^ Diagnóstico de Síndrome de Edwards 13 dias após o procedimento. ^2^ Realizada valvoplastia pulmonar no mesmo procedimento.

**Tabela 2:** Dados do procedimento

< diâmetro (mm) canal arterial	> diâmetro (mm) canal arterial	Comprimento	Dispositivo	Sucesso	Shunt residual imediato	Shunt residual 7 dias	Complicações maiores	Obstrução APE leve	Extubação (dias)
2	3	5	ADOII AS 0402	Sim	Discreto	Não	Não	Não	7
2,3	3,5	6	ADOII AS 0402	Sim	Não	Não	Não	Sim	Não
2	4	4,8	ADOII AS 0402	Sim	Não	Não	Não	Não	12
2,5	5,6	6,5	ADOII AS 0402	Sim	Não	Não	Não	Não	5
3,5	4,5	5,5	ADOII AS 0504	Sim	Não	Não	Não	Não	25
2	3,2	6	ADOII AS 0402	Sim	Não	Não	Não	Não	15
3	5	8	ADOII AS 0402	Sim	Não	Não	Não	Não	7
3,5	4	12	ADOII AS 0406	Sim	Sim	Não	Não	Sim	23
3,5	5	10	PICCOLO 0402	Sim	Não	Não	Não	Não	
3	4	7	ADOII AS 0502	Sim	Não	Não	Não	Não	
3,5	3,8	6	ADOII AS 0502	Sim	Não	não	Não	Não	Não
3,8	3,8	4	PICCOLO 0502	Sim	Não	Não	Não	Não	7
3,9	4,2	8	ADOII AS 0502	Sim	Não	não	Não	Não	
3,5	3,5	9	PICCOLO 0502	Sim	Não	Não	Não	Não	22
3,00	4,08	6,99		100%	14%	0%	0%	14%	13,6

*APE: artéria pulmonar esquerda*

Ambos os pacientes que necessitavam de drogas vasoativas tiveram suspenso o uso em 24 horas após o procedimento. Não houve relato de instabilidade hemodinâmica após o procedimento. Não houve complicações maiores durante ou após o procedimento. Em dois pacientes foi diagnosticada estenose leve da artéria pulmonar esquerda relacionada ao dispositivo, sem significância clínica.

## Discussão

Pacientes prematuros têm incidência aumentada de canal arterial patente devido a diversos fatores, como maior sensibilidade dos receptores de prostaglandinas e maior exposição à hipóxia e à acidose tecidual. Historicamente, o fechamento do canal arterial no prematuro é realizado pela administração de anti-inflamatórios não esteroides ou por cirurgia aberta, abordagens com limitações e não isentas de complicações. A primeira descrição de oclusão percutânea de canal arterial em prematuro ocorreu em 2005 em um paciente de 1400 gramas que foi submetido a fechamento com mola “Flipper,”^11^ ” Em 2007, Roberts P. et al.,^12^ descreveram o fechamento percutâneo de canal arterial em 10 pacientes bem selecionados com peso entre 1660 e 2600 gramas utilizando novamente molas “Flipper”.^12^ Molas de liberação controlada não foram desenvolvidas para fechamento de canais arteriais grandes e algumas vezes são necessários 2 ou 3 dispositivos para ocluir um canal arterial de 3 ou 4 mm. Francis et al.,^13^ descreveram o fechamento percutâneo de canal arterial em pacientes prematuros com peso médio de 1100 g utilizando uma técnica específica de implante simultâneo de 2 ou 3 molas.^13^ Entretanto, apenas 10% dos pacientes dessa instituição tinham anatomia favorável para o fechamento com molas mostrando a limitação desta técnica nessa população. A forma característica e uniforme do canal arterial do prematuro também não favorece o fechamento com dispositivos tradicionais com ADO I (Abbot Structural Heart, Plymouth, MN) ou ADO II (Abbot Structural Heart, Plymouth, MN) devido ao tamanho dos discos que determinam obstrução aos fluxos aórtico e/ou pulmonar.^14 - 16^ O advento do dispositivo ADO II AS (Abbot Structural Heart, Plymouth, MN), com discos apenas 1 mm maiores que o centro, permitiu o fechamento percutâneo de pacientes abaixo de 3 kg com segurança, por via venosa e sem as complicações previamente descritas com dispositivos maiores.^17^ O primeiro estudo com pacientes abaixo de 1 kg demonstrou resultados promissores com fechamento por via venosa, sem instabilidade hemodinâmica ao cruzar a valva tricúspide com guia e sistema de liberação de baixo perfil e sem complicações.^18^ Em 2020, um grande estudo francês com 102 pacientes, 21 deles abaixo de 1 kg, confirmou os excelentes resultados dessa técnica e mostrou que a imensa maioria dos pacientes dessa população de prematuros se beneficiam dos dispositivos com apenas 2 mm de comprimento.^19^ Por fim, em estudo desenhado para aprovação do dispositivo no FDA, 100 pacientes prematuros foram submetidos a fechamento percutâneo com o dispositivo Piccolo ^tm^(Abbot Structural Heart, Plymouth, MN), especificamente desenvolvido para fechamento percutâneo de canal arterial de prematuros, com resultados novamente animadores, sem shunt residual em 6 meses e sem casos de obstrução aórtica ou pulmonar significativas relacionadas ao procedimento.^20^ A experiência brasileira vai de encontro com a literatura. São 14 casos em pacientes abaixo de 2 kg, com 100% de taxa de oclusão do dispositivo em 48 horas e sem complicações maiores. Apenas 2 pacientes apresentaram estenose não significativa de ramo pulmonar esquerdo que não foi clinicamente significativa. Um desses pacientes, no início da experiência, com canal arterial longo (12 mm), foi submetido a fechamento com dispositivo de 6 mm de comprimento e acredita-se que o comprimento desse dispositivo esteja relacionado à estenose de artéria pulmonar esquerda. Todos os pacientes, desde então, receberam dispositivos com 2 mm de comprimento, exceto por 1 paciente que recebeu um dispositivo com 4 mm de comprimento. Nenhum paciente necessitou de transfusão sanguínea devido a sangramento importante, e todos evoluíram favoravelmente do ponto de vista ventilatório com desmame em média após 13,6±7,4 dias do procedimento. Não houve mortes relacionadas ao procedimento; entretanto, 3 pacientes não sobreviveram ao final do estudo devido a outras causas, mostrando que se trata de uma população grave com altas taxas de mortalidade.

Nas últimas décadas, um extenso debate tem sido realizado com o objetivo de avaliar os benefícios do fechamento do canal arterial em pacientes prematuros. O uso de anti-inflamatórios não esteroides ainda hoje é a primeira opção terapêutica, porém está associado a maior incidência de lesão renal, enterocolite necrotizante, além de ter baixa efetividade. Já os pacientes submetidos ao fechamento cirúrgico têm risco aumentado de diminuição do débito cardíaco, hipoperfusão sistêmica e lesão cerebral no pós-operatório, além de estar associado a maior incidência de displasia broncopulmonar e retinopatia da prematuridade no seguimento tardio.^21 - 23^ Inúmeros estudos tiveram dificuldade em comprovar os benefícios do tratamento do canal arterial do prematuro com anti-inflamatórios não esteroides ou cirurgia, resultando em importante diminuição da indicação nos centros de neonatologia nos Estados Unidos e ao redor do mundo.^24^ Essa mudança de conduta, aparentemente, pode ter contribuído para o pior desfecho desses pacientes como demonstrado recentemente por estudo comparando duas amostras de períodos diferentes em um grande centro de neonatologia dos Estados Unidos.^25^ Quando comparado o fechamento cirúrgico com o percutâneo nos pacientes prematuros, houve uma melhora mais rápida no padrão respiratório nos pacientes submetidos ao procedimento por cateterismo, além de uma menor taxa de complicações associadas ao procedimento.^26^ Nesse contexto, o advento de uma terapêutica de baixo risco é essencial para evitar o desenvolvimento de danos relacionados ao baixo débito prolongado a que são submetidos alguns pacientes prematuros, e que claramente estão associados ao desenvolvimento de enterocolite necrotizante, displasia broncopulmonar e hemorragia intraventricular. Atualmente, o fechamento percutâneo do canal arterial de pacientes prematuros acima de 700 gramas é um procedimento seguro, com alta eficácia, baixíssima taxa de complicações e está comprovadamente associado à melhora do prognóstico de pacientes bem selecionados. A maioria dos pacientes deste estudo tiveram melhora dos parâmetros ventilatórios após o fechamento do canal arterial. Resta a dúvida quanto ao melhor momento do procedimento. O alto índice de morbidade do tratamento clínico com anti-inflamatórios não esteroides pode fazer com que, num futuro próximo, o fechamento percutâneo do canal arterial seja a primeira escolha em pacientes prematuros bem selecionados com repercussão hemodinâmica, sobrecarga de câmaras esquerdas e risco de complicações da prematuridade associado ao canal arterial.

### Limitações

A principal limitação do presente estudo foi a falta de padronização da indicação do fechamento do canal arterial entre os centros envolvidos. A indicação do fechamento do canal arterial em pacientes prematuros tem sido extensamente debatida nas últimas décadas e ainda não há consenso entre neonatologistas sobre os critérios para utilização de anti-inflamatórios não esteroides, cirurgia ou até mesmo o fechamento percutâneo. Esse contexto dificulta muito a padronização de critérios de indicação entre diferentes centros de um país continental como o Brasil. Estudos como este, ainda que com número limitado de pacientes, são extremamente importantes para apresentação da segurança do procedimento e dos resultados imediatos. Novos estudos com detalhamento das indicações do procedimento serão fundamentais para a definição dos pacientes que mais se beneficiam dessa técnica. Estudos de evolução de longo prazo também devem ser conduzidos para o adequado seguimento destes pacientes por se tratar de estratégia nova implantada na condução desses pacientes.

## Conclusão

O fechamento percutâneo do canal arterial em pacientes prematuros abaixo de 2 kg é uma realidade em nosso país e está associado a melhora dos parâmetros ventilatórios, na imensa maioria dos pacientes incluídos neste estudo. Além disso, é um procedimento eficaz e extremamente seguro, com baixíssima taxa de complicações menores. A população que provavelmente mais se beneficia desse procedimento é composta de pacientes graves com alta morbimortalidade. O desenvolvimento de um procedimento seguro e eficaz pode significar um avanço no tratamento do canal arterial patente do paciente prematuro.
