# Supplementary material for: Fechamento Percutâneo do Canal Arterial em Pacientes Prematuros Abaixo de 2 Kg: Experiência Inicial Brasileira
Source: Arq Bras Cardiol. 2022 Aug 25;119(3):460–7. [Article in Portuguese] doi: 10.36660/abc.20210818 (PMC9438535; doi:10.36660/abc.20210818)
Supplement: Translation

# Translation: Percutaneous Closure of Ductus Arteriosus in Preterm Babies: The Initial Brazilian Experience

**Authors:** João Luiz Langer Manica, Juliana Rodrigues Neves, Raul Arrieta, Pedro Abujamra, Raul Ivo Rossi, Luiz Carlos Giuliano, Germana Coimbra, Pablo Tomé Teixeirense, João Henrique Aramayo Rossi, Rodrigo Nieckel da Costa, Salvador André Bavaresco Cristóvão, Carlos Pedra

**Affiliations:** 1 Brazil Instituto de Cardiologia/Fundação Universitária de Cardiologia (IC/FUC), Porto Alegre, RS – Brazil; 2 Brazil Real Hospital Português de Beneficência em Pernambuco, Recife, PE – Brazil; 3 Brazil Hospital Sepaco, São Paulo, SP – Brazil; 4 Brazil Santa Casa de São José dos Campos, São José dos Campos, SP – Brazil; 5 Brazil Hospital de Clínicas da UNICAMP, Campinas, SP – Brazil; 6 Brazil Universidade Federal de Santa Catarina, Florianópolis, SC – Brazil; 7 Brazil Hospital SOS Cárdio, Florianópolis, SC – Brazil; 8 Brazil Hospital Unimed de Piracicaba, Piracicaba, SP – Brazil; 9 Brazil Pontifícia Universidade Católica do Rio Grande do Sul, Porto Alegre, RS – Brazil; 10 Brazil Instituto Dante Pazzanese de Cardiologia, São Paulo, SP – Brazil; 11 Brazil Hospital e Maternidade Santa Joana, São Paulo, SP – Brazil; 12 Brazil Beneficência Portuguesa de São Paulo, São Paulo, SP – Brazil; 13 Brazil Hospital do Coração, São Paulo, SP – Brazil

**Keywords:** Heart Defects, Congenital, Ductus Arteriosus, Catheterization, Infant, Premature, Neonatology

## Abstract

**Background:**

The presence of patent ductus arteriosus can be as high as 50% in preterm babies. Hemodynamically significant patent ductus arteriosus is a common cause of delayed weaning of respiratory support and an important risk factor of necrotizing enterocolitis, intraventricular hemorrhage, and bronchopulmonary dysplasia in this population.

**Objective:**

The aim of this study is to describe an initial experience of percutaneous closure of the ductus arteriosus in preterm infants weighing less than 2 kg.

**Methods:**

This was a prospective study, comprised of 14 consecutive patients submitted to percutaneous closure of ductus arteriosus between March 2020 and February 2021 in 6 institutions in Brazil.

**Results:**

Mean gestational age was 28.45±3.14 weeks, mean age at the procedure was 38.85±17.35 days and mean weight was 1.41 ±0.41 kg; 79% of the patients were under mechanical ventilation, and 79% had been submitted, on average, to a 1.5 cycle of non-steroidal anti-inflammatory drugs. Most patients were weaned off of mechanical ventilation in a mean of 12.6 ±7.24 days after the procedure. Success rate was 100%. No procedure-related mortality was observed.

**Conclusion:**

This study concluded that percutaneous closure of ductus arteriosus in premature babies below 2 kg has satisfactory results and a low complication rate in this study sample.

## Introduction

The incidence of patent *ductus arteriosus* (PCA) may reach 50% in premature patients. When hemodynamically significant, it can be responsible for extended mechanical ventilation time, in addition to being an important risk factor for necrotizing enterocolitis, intraventricular hemorrhage, and bronchopulmonary dysplasia in this population.^1 - 5^ Some patients benefit from PCA closure in this period of life, with an important progression in ventilatory weaning and improvement of the overall outcome. Historically, the gold standard treatment has been medicinal therapy with non-steroidal anti-inflammatory drugs, even with success rates around 60% and associated with significant adverse effects.^6^ Surgical ligation is an alternative for patients who do not have the conditions for an enteral diet or after failure of the medicinal therapy. However, up to 45% of the patients develop hemodynamic instability shortly after the surgical procedure.^7 - 10^ Until 2010, only a few isolated cases of percutaneous closure of ductus arteriosus in preterm babies had been reported in the literature. The arrival of the Amplatzer Duct Occluder II Additional Sizes (ADO II AS) (Abbot Structural Heart, Plymouth, MN) device revolutionized PCA treatment in preterm patients weighing less than 2kg. More recently, the Piccolo^tm^(Abbot Structural Heart, Plymouth, MN) device was designed specifically for that population and approved by FDA. The purpose of this study is to describe the initial experience of the percutaneous closure of ductus arteriosus in premature infants weighing less than 2 kg.

## Methods

This is a prospective study about the percutaneous treatment of ductus arteriosus in newborn premature infants with ≤ 2 kg of weight, conducted with new devices dedicated to that population. The procedures were conducted in six centers with distinct operators in the period from March 2020 to February 2021. All agreed to participate in this study. Patients were selected based on specific criteria of each center involved in the study. However, all patients whose enteral nutrition was not contraindicated had received oral treatment for ductus arteriosus closure with an unsuccessful application of at least one cycle of non-steroidal anti-inflammatory drugs before recommending the percutaneous procedure. Furthermore, the need for refractory extended mechanical ventilation, associated with the presence of patent ductus arteriosus with signs of volumetric overload and an increase of the left atria, was the main indication for the use of non-steroidal anti-inflammatory drugs and, subsequently, percutaneous closure of ductus arteriosus should the clinical treatment fail.

Collected data: demographic data: gestational age, birth weight, gender, age (in days), and weight (in g) at the moment of the procedure; clinical data: use of mechanical ventilation and vasoactive drugs, associated comorbidities, use of previous drugs for ductus arteriosus closure (ibuprofen, paracetamol and others) and procedure data: vascular access route, device type and size, use of contrast, technical difficulties reported and related complications, such as: pulmonary artery or aorta stenosis, among others. Post-procedure data and evolution, such as the presence of residual shunt, mechanical ventilation and vasoactive drugs weaning, and cardiac function were also recorded.

Statistical Analysis: The quantitative variables were described through mean and standard deviation, as there was no normality violation, assessed through the Kolmogorov-Smirnov Test at the significance level of 5%. The categorical variables were described through absolute (n) and relative (%) frequencies. The SPSS software, version 21 was used.

This study was approved by the Ethics Committee on Research of the Cardiology Institute of RS and follows resolution 466/2012. All patients’ legal guardians signed the free and informed consent form.

### Description of device and technique

All procedures were conducted in the catheterization laboratory or surgery room, using the C-arm of the respective services, requiring the transportation of the newborn to that sector. The procedure was conducted under general anesthesia. Care with temperature maintenance was taken, with minor variations among the centers and, in general, involving the use of heated mattress or blankets, temperature monitoring with rectal or esophageal thermometer, and/or extra heating by covering the ends and cephalic pole. To reduce the procedure time, blood loss, and delivery of unnecessary fluids, invasive blood pressure measurements were not performed routinely. Puncture of the femoral vein was performed with a 21G needle or a 22G jelco and guided by vascular ultrasonography. A 4F radial introducer was then inserted. A JR curve or vertebral (Cordis or Terumo) catheter (4F) guided by a 0.014” flexible guide of moderate support was, then, placed through the right chambers and the ductus arteriosus in the descending aorta ( Figure 1A ). For placing the prothesis delivery system (4Fr TorqVue, Abbot Structural Heart), different supporting techniques can be used, namely: the use of microcatheter on a 0.014” guide previously placed in the descending aorta ( Figure 1B ), replacing the 0.014” guide with a 0.035” teflonized guide placed in the descending aorta, use of the 0.014” guide placed in the contralateral femoral artery and pressed externally, or use of two 0.014” parallel guides to increase the support. To preserve the renal functions of such premature babies, the use of contrast was limited to small manual injections, only when necessary, in order to clarify uncertainties regarding the procedure in some cases ( Figure 2 ). All procedures were guided by transthoracic echocardiography to measure the ductus arteriosus and to place and release the device ( Figure 3A and B ).

**Figure 1:** A) 0.014” guide crossing the ductus arteriosus inserted through a catheter placed in the right ventricle. B) Microcatheter on 0.014” guide crossing the ductus arteriosus and serving as support to access the delivery system of the device.

**Figure 2:** Injection of contrast in a 90° position through the release system.

**Figure 3:** A) Echocardiogram performed during the procedure, with measures of the diameters in the aortic and pulmonary ends and ductus length. B) Echocardiogram performed immediately after the release of the device so as to dismiss residual injury such as left pulmonary artery or aorta stenosis.

The Amplatzer ADO IIAS and Piccolo (Abbot Structural Heart, Plymouth, MN) devices, both developed for the occlusion of ductus arteriosus in small children, the latter being specific for premature newborn infants weighing more than 700g and commercially available in Brazil from mid-2020, were used in all procedures. Both present similar features regarding structure, as they consist of compact nitinol mesh to minimize the residual shunt immediately after the implant, symmetrical design composed of two articulated discs, and a central belt corresponding to the dimensions of the low-profile device with lengths of 2, 4, and 6 mm, in addition to the delivery system and cable, also flexible to facilitate placing and release ( Figure 4A and 4B ). The devices were selected to be at least 1 mm larger than the ductus arteriosus in diameter and with a shorter length than the ductus arteriosus to prevent pulmonary artery or aorta stenosis. As previously mentioned, prothesis placing and release were guided by echocardiography, in addition to fluoroscopy, observing the presence of residual shunt or stenosis in the left branch of the pulmonary artery or aorta caused by the device. If present, the device may be replaced before its full release. After the procedure, the patients are transported in a heated incubator back to the neonatal ICU.

**Figure 4:** A) TorqueVue 4F delivery system and device. B) 4 mm diameter PiccoloTM device in different lengths 2, 4, and 6 mm.

## Results

From March 2020 to February 2021, percutaneous closure of ductus arteriosus in premature infants weighing less than 2 kg was performed in 14 patients by 8 different operators in Brazil. Demographic data are described in Table 1 . The average age of the patients at the moment of the procedure was 38.85 ± 17.35 days and the average weight during the procedure was 1.41 ± 0.41Kg. Four patients weighed < 1 kg at the moment of the procedure. Most of the patients needed mechanical ventilation during the procedure (11/14) and at least 6 patients had bronchopulmonary dysplasia diagnosis. Three patients had received no previous cycle of ibuprofen, one due to acute kidney injury and anuria, one due to duodenal atresia, and one due to a tracheoesophageal fistula. The recommendation of the closure of ductus arteriosus was defined by the neonatology team of each institution. Data on the procedure are described in Table 2 . The average diameter of ductus arteriosus was around 3.0 0.67 mm, and the average length was 6.9 ± 2.12 mm. No patient was submitted to puncture in the artery. Heparinization after venipuncture was not performed routinely and depends on the operator’s choice. The most used device was 0402 ADO II AS or Piccolo (Abbot Structural Heart, Plymouth, MN) in 7 cases, followed by 0502 ADO II AS or Piccolo (Abbot Structural Heart, Plymouth, MN) in 5 patients. In one patient, the ADO II AS 0504 device (Abbot Structural Heart, Plymouth, MN) was implemented, while in another one, the ADO II AS 0406 device (Abbot Structural Heart, Plymouth, MN) was implanted, both longer than the others. One patient needed pulmonary valvuloplasty during the procedure due to pulmonary valve stenosis. Two patients presented a drop in systemic saturation related to tricuspid insufficiency during the procedure. No patient presented significant tricuspid regurgitation after the procedure. The success rate of the procedure was 100%. Two patients presented residual shunt immediately after the procedure, however, in 100% of the cases there was no residual shunt in 7 days. The three patients depending on nasal oxygen catheter discontinued the use in an average of three days after the procedure. Amongst the patients using oxygen catheter, one of them had the closure of ductus arteriosus recommended due to overt cardiac failure, with a weight lower than the birth weight after 55 days of life, with use of a nasogastric tube and a clinical picture of important malnutrition. This patient experienced an immediate improvement, returning to oral administration immediately after extubation and discharge after 48 hours in an excellent clinical condition. From the 11 patients in mechanical ventilation, nine patients were extubated within an average of 13.6 ± 7.4 days after the procedure. No complications related to vascular access were reported. Three deaths were reported among the patients in the study, none related to the procedure. One patient improved therenal function, experiencing a return of diuresis two days after the procedure, with an improvement in ventilatory parameters but not evolving to extubation due to a broad intraventricular communication and Edwards syndrome diagnosed 13 days after the procedure. That patient evolved to death by sepsis not related to the procedure after 22 days. Another patient with associated genetic syndrome, bronchopulmonary dysplasia, and severe pulmonary artery hypertension had also not achieved the clinical conditions for extubation and evolved to death 30 days after the procedure due to sepsis. The other patient was extubated 23 days after the procedure, experienced great evolution, and 57 days after the procedure, weighing approximately 2 kg, became infected with SARS-CoV-2 (COVID-19), evolving again to mechanical ventilation and extubation 10 days later. That patient, 80 days after the procedure, weighing around 2300g, using O_2_through nasal catheter at 0.5 L/min due to bronchopulmonary dysplasia, presented sudden mesenteric ischemia, was submitted to urgent surgery, and evolved to death. In this case, it is not possible to dismiss post-COVID-19 thrombosis.

**Table 1:** Demographic data

GA (weeks)	BW	Age (days)	Weight (Kg)	MV^*^	VAD^†^	NSAID cycles^‡^	Comorbidities
28.6	0.58	62	0.965	Yes	No	1	BDP^§^, HIC^//^
29.2	1.01	15	0.92	Yes	Yes	No	AKI^¶^in anuria, Genetic syndrome^1^, wide IVC^#^
26.6	0.82	41	1.15	Yes	No	2	BPD, ICH, exposed B24
32	1.55	33	1.85	Yes	No	2	Suspected dandy-walker
26	0.85	45	1.55	Yes	No	3	BPD
27	0.7	28	1.2	Yes	No	2	
26	0.9	32	1.5	Yes	No	2	^**^PVS^2^
25	0.8	45	1.2	Yes	Yes	2	BPD
35	2	55	1.95	No	No	1	
35	1.2	58	2.0	No	No	1	
28	0.8	45	1.45	Yes	Yes	1	BPD, severe PH^††^, Genetic syndrome
27	0.98	7	0.98	Yes	No	No	duodenal atresia
26	0.58	63	2.0	No	No	2	BPD
27	0.78	15	0.96	Yes	Yes	No	tracheoesophageal fistula, gastrostomy
28.46	0.97	38.86	1.41	79%	28%	1.55	

GA: gestational age; BW: birth weight;^*^MV: mechanical ventilation;^†^VAD: vasoactive drug;^‡^NSAID: non-steroidal Anti-inflammatory drug;^§^BPD: bronchopulmonary dysplasia;^//^ICH: intracranial hemorrhage;^¶^AKI: acute kidney injury;^#^IVC: interventricular communication;^**^PVS: pulmonary valve stenosis;^††^PH: pulmonary hypertension.^1^Diagnosis of Edwards Syndrome 13 days after the procedure.^2^Pulmonary valvuloplasty performed in the same procedure.

**Table 2:** Procedure data

ductus arteriosus < diameter (mm)	ductus arteriosus > diameter (mm)	Length	Device	Success	Immediate residual shunt	7-day residual shunt	Major complications	Mild LPA obstruction	Extubation (days)
2	3	5	ADOII AS 0402	Yes	Discreto	No	No	No	7
2.3	3.5	6	ADOII AS 0402	Yes	No	No	No	Yes	No
2	4	4.8	ADOII AS 0402	Yes	No	No	No	No	12
2.5	5.6	6.5	ADOII AS 0402	Yes	No	No	No	No	5
3.5	4.5	5.5	ADOII AS 0504	Yes	No	No	No	No	25
2	3.2	6	ADOII AS 0402	Yes	No	No	No	No	15
3	5	8	ADOII AS 0402	Yes	No	No	No	No	7
3.5	4	12	ADOII AS 0406	Yes	Yes	No	No	Yes	23
3.5	5	10	PICCOLO 0402	Yes	No	No	No	No	
3	4	7	ADOII AS 0502	Yes	No	No	No	No	
3.5	3.8	6	ADOII AS 0502	Yes	No	No	No	No	No
3.8	3.8	4	PICCOLO 0502	Yes	No	No	No	No	7
3.9	4.2	8	ADOII AS 0502	Yes	No	No	No	No	
3.5	3.5	9	PICCOLO 0502	Yes	No	No	No	No	22
3.00	4.08	6.99		100%	14%	0%	0%	14%	13.6

*LPA: left pulmonary artery.*

Both patients who needed vasoactive drugs discontinued the use 24 hours after the procedure. No hemodynamic instability was reported after the procedure. No major complications were reported during or after the procedure. Two patients were diagnosed with mild stenosis in the left pulmonary artery related to the device, with no clinical significance.

## Discussion

Premature infant patients have an increased incidence of patent ductus arteriosus due to several factors, including greater sensitivity of prostaglandin receptors and greater exposure to hypoxia and tissue acidosis. Historically, closure of ductus arteriosus in premature infants is performed through the administration of non-steroidal anti-inflammatory drugs or by open surgery, approaches that are limited and not exempt from complications. The first description of percutaneous occlusion of ductus arteriosus in premature infants occurred in 2005 in a patient weighing 1400 grams, who was subjected to closure using “Flipper” coil.^11^ In 2007, Roberts P et al.^12^ described the percutaneous closure of ductus arteriosus in 10 well-selected patients weighing between 1660 and 2600 grams, once again using “Flipper” coils.^12^ Controlled release coils were not developed for the closure of large ductus arteriosus, and often 2 or 3 devices are required to occlude a 3- or 4-mm ductus arteriosus. Francis et al.^13^ described percutaneous closure of ductus arteriosus in premature infant patients with an average weight of 1100 g using a specific technique of the simultaneous implant of 2 or 3 coils.^13^ However, only 10% of the patients of this institution had favorable anatomy for closure with coils, showing the limitation of that technique in this population. The characteristic and uniform shape of the premature infant’s ductus arteriosus also does not favor closure with traditional devices like ADO I (Abbot Structural Heart, Plymouth, MN) or ADO II (Abbot Structural Heart, Plymouth, MN) due to the size of the discs that determine obstruction to aortic and/or pulmonary flows.^14 - 16^ The arrival of the ADO II AS (Abbot Structural Heart, Plymouth, MN) device, with discs only 1 mm larger than the center, allowed safe intravenous percutaneous closure for patients under 3 kg, without the complications previously described with larger devices.^17^ The first study with patients under 1 kg displayed promising results with percutaneous closure, with no hemodynamic instability when crossing the tricuspid valve, with a guide and low-profile delivery system and without complications.^18^ In 2020, a large French study with 102 patients, 21 of whom under 1 kg, confirmed the excellent results of this technique and showed that most patients in that population of premature infants benefited from devices with only 2 mm of length.^19^ Finally, in a study designed for the approval of the device by FDA, 100 premature infant patients were subjected to percutaneous closure with the Piccolo^tm^(Abbot Structural Heart, Plymouth, MN) device, specifically developed for percutaneous closure of ductus arteriosus in premature infants, once again with encouraging results, with no residual shunt in 6 months and without cases of significant aortic or pulmonary obstruction related to the procedure.^20^ The Brazilian experience meets literature. It involves 14 cases of patients under 2 kg, with a device occlusion rate of 100% in 48 hours and with no major complications. Only two patients presented non-significant stenosis in the left pulmonary branch, which were not clinically significant. One of these patients, in the beginning of the experience, with long ductus arteriosus (12 mm), was subjected to closure with a 6-mm device, and it is believed that the length of that device is related to the stenosis of the left pulmonary artery. All patients since then have received 2-mm-long devices, except for 1 patient who received a 4-mm-long device. No patient needed blood transfusion due to important bleeding, and all of them evolved favorably from a ventilatory point of view with weaning in an average of 13.6 ± 7.4 days after the procedure. No death related to the procedure was reported, however, three patients did not survive at the end of the study due to other causes, showing that this is a severe population with high mortality rates.

In recent decades, an extensive debate has taken place with the purpose of assessing the benefits of the closure of ductus arteriosus in premature infant patients. The use of non-steroidal anti-inflammatory drugs is still the first therapeutic option today, but it is associated with a greater incidence of renal injury, necrotizing enterocolitis, in addition to low effectiveness. By contrast, patients submitted to surgical closure have an increased risk of low cardiac output, systemic hypoperfusion, and brain damage after the surgery, in addition to being associated with a greater incidence of bronchopulmonary dysplasia and retinopathy of prematurity in long-term follow-up.^21 - 23^ Countless studies faced difficulties to prove the benefits of treating the ductus arteriosus of premature infants with non-steroidal anti-inflammatory drugs or surgery, resulting in an important decrease in recommendations in neonatology centers in the United States and around the world.^24^ This change of approach may have contributed to the worst outcome of such patients, as shown recently by a study comparing two samples from different periods in a large neonatology center in the United States.^25^ Comparing surgical closure with percutaneous closure in premature infant patients, a faster improvement in the respiratory pattern of patients submitted to percutaneous approach was noted, in addition to a lower rate of complications associated with the procedure.^26^ In that context, the arrival of a low-risk therapy is essential to prevent the development of damages related to extended low output which some premature infant patients suffer from, and that are clearly associated with the development of necrotizing enterocolitis, bronchopulmonary dysplasia, and intraventricular hemorrhage. Currently, percutaneous closure of ductus arteriosus in premature infant patients over 700 grams is a safe procedure, with high effectiveness and exceptionally low complication rates, and it has been proven to be associated with the improvement of the prognosis for well-selected patients. Most of the patients in this study experienced improvement in ventilatory parameters after the closure of ductus arteriosus. One doubt remains regarding the best moment for the procedure. The high morbidity rate of the clinical treatment with non-steroidal anti-inflammatory drugs may cause, in the near future, the percutaneous closure of ductus arteriosus to be the first choice for well-selected premature infant patients with hemodynamic repercussion, left chamber overload, and the risk of prematurity complications associated with ductus arteriosus.

### Limitations

The main limitation of this study was the lack of standardization of the recommendation for the closure of ductus arteriosus among the participating centers. Recommendation of closure of ductus arteriosus in premature infant patients has been extensively discussed in recent decades, and still no consensus has been reached among neonatologists regarding the criteria for use of non-steroidal anti-inflammatory drugs, surgery, or even percutaneous closure. This context makes it much more difficult to standardize the recommendation criteria among the different centers in a continental country such as Brazil. Studies such as this, even with a limited number of patients, are extremely important to show the safety of the procedure and the immediate results. Novel studies detailing the indications of the procedure are warranted to define the patients who benefit the most from this technique. Long-term evolution studies must be conducted as well, for an adequate follow-up of the patients, given that it is a new strategy in the management of such patients.

## Conclusion

According to the sample presented, percutaneous closure of ductus arteriosus in premature infant patients under 2 kg is associated with the improvement of ventilatory parameters in most of the patients included in this study. Furthermore, it is an effective and extremely safe procedure, with an exceptionally low rate of minor complications. The population that benefits the most from this procedure consists of severe patients with high morbimortality. The development of a safe and effective procedure may mean an advance in the treatment of patent ductus arteriosus in premature infant patients.
